# Treatment of oral mucositis due to chemotherapy

**DOI:** 10.4317/jced.52917

**Published:** 2016-04-01

**Authors:** Begonya Chaveli-López, José V Bagán-Sebastián

**Affiliations:** 1 DDS. Stomatology Department, Faculty of Medicine and Dentistry, University of Valencia, Spain; 2 MD, DDS, PhD. Head of the Department of Stomatology and Maxillofacial Surgery. Chairman of Oral Medicine. University of Valencia, Spain

## Abstract

**Introduction:**

The management of oral mucositis is a challenge, due to its complex biological nature. Over the last 10 years, different strategies have been developed for the management of oral mucositis caused by chemotherapy in cancer patients.

**Material and Methods:**

An exhaustive search was made of the PubMed-Medline, Cochrane Library and Scopus databases, crossing the key words “oral mucositis”, “prevention” and “treatment” with the terms “chemotherapy” and “radiotherapy” by means of the boolean operators “AND” and “NOT”. A total of 268 articles were obtained, of which 96 met the inclusion criteria.

**Results:**

Several interventions for the prevention of oral mucositis, such as oral hygiene protocols, amifostine, benzidamine, calcium phosphate, cryotherapy and iseganan, among others, were found to yield only limited benefits. Other studies have reported a decrease in the appearance and severity of mucositis with the use of cytoprotectors (sucralfate, oral glutamine, hyaluronic acid), growth factors, topical polyvinylpyrrolidone, and low power laser irradiation.

**Conclusions:**

Very few interventions of confirmed efficacy are available for the management of oral mucositis due to chemotherapy. However, according to the reviewed literature, the use of palifermin, cryotherapy and low power laser offers benefits, reducing the incidence and severity of oral mucositis – though further studies are needed to confirm the results obtained.

** Key words:**Chemotherapy-Induced Oral Mucositis Treatment.

## Introduction

The term “mucositis” was introduced in late 1980 to describe inflammation of the oral mucosa induced by radiotherapy (observed in 80% of the patients), chemotherapy (in 40-80% of the patients) and bone marrow transplantation (in over 75% of the patients) – the phenomenon being regarded as a manifestation of leukopenia (1-3). At present, oral mucositis is considered to be the most serious non-hematological complication of cancer treatment (3).

-Physiopathology of mucositis

While it is clear that chemotherapeutic agents target rapidly dividing healthy tissues, including the oral mucosa and gastrointestinal tract, new studies indicate that damage to submucosal components occurs before the epithelial lesions become manifest (4,5). Specifically, damage and apoptosis of fibroblasts and vascular endothelial cells appear to precede the epithelial lesions. On a mechanical basis, endothelial cell damage resulting from the loss of secretion of epithelial growth factors such as keratinocyte growth factor (KGF) may explain the deregulation of the normal mucosal epithelial growth patterns (2,4). Other critical factors leading to ulceration include the early release of inflammatory cytokines and reactive oxygen species (ROS) at mucosal level. This in turn activates transcription factors such as nuclear factor kappa B (NF-kB), inducing the over-regulation of specific genes (tumor necrosis factor, IL-6, and IL-1) and triggering apoptosis and the cascade of events leading to epithelial ulceration (2,6,7). It is also believed that bacterial colonization and/or secondary colonization of the ulcers prolongs the corresponding healing times (2). However, recent hypotheses and clinical data suggest that infection is not a central element in the development of mucositis (5-7).

-Risk factors for mucositis

A number of risk factors have been reported to influence the frequency and severity of mucositis. Some factors are related to the patient, such as the type of tumor involved (hematological diseases) (1-3), age (young patients) (3), buccodental health (poor oral hygiene before and during chemotherapy) (3), the nutritional condition of the patient, and the maintenance of kidney and liver function. Other factors are related to the type of cytostatic agent used, e.g., drugs that affect DNA synthesis, such as antimetabolites (methotrexate, 5-fluorouracil) and purine analogs (cytarabine), are associated to incidences of oral mucositis of close to 40-60% (4). Furthermore, methotrexate and etoposide are secreted in saliva, which increases their oral toxicity. However, asparaginase and carmustine are not related to the development of mucositis (2). With regard to combinations of cytostatic agents, between 40-70% of all patients receiving standard chemotherapy regimens develop mucositis (2,3). Lastly, the frequency of administration and concomitant treatment with radiotherapy and/or bone marrow transplantation are also factors that condition the appearance of mucositis (3,4,7).

-Clinical characteristics of mucositis

Mucositis manifests as erythema, edema or ulceration that can be accompanied by alterations ranging from mild burning sensation to large and painful ulcers that worsen patient quality of life and limit basic oral functions such as speech, the swallowing of saliva or eating (2,4). Mucositis tends to appear sooner after chemotherapy than after radiotherapy, and more often affects the non-keratinized mucosa (2). Its maximum expression occurs 7-10 days after chemotherapy, and erythema progresses towards ulceration. This is the period of maximum patient pain and discomfort, and in many cases requires the administration of opioids and changes in diet. Mucositis then gradually subsides, leaving no scars, over a period of 2-3 weeks after infusion of the drug, provided the patient does not present bone marrow suppression. The development of infections, caused mainly by herpes simplex virus or *Candida albicans* (though other species of *Candida*, such as *krusei tropicalis*, *parapsilosis* and *glabrata*, or other fungal genera such as *Aspergillus* and *Mucor*, may also be involved), is a serious complication observed mainly in patients with prolonged neutropenia, and may prove life-threatening (2). *Streptococcus oralis* and *Streptococcus mitis* are among the most common bacteria isolated from blood, and *S. mitis* can cause adult respiratory distress syndrome, particularly when high-dose cytarabine is administered (2).

-Evaluation

A number of methods have been developed to measure and quantify the changes that occur in the oral epithelium. However, the scale most commonly used in research is that developed by the World Health Organization (WHO), which combines the clinical characteristics of the oral mucosa with the capacity of the patient to eat (1-3,5). As regards the clinical evaluation of mucositis, the most widely used scale is that forming part the Common Toxicity Criteria for Adverse Events (CTCAE) of the United States National Cancer Institute, which contemplate the patient symptoms, the capacity for oral intake, and the need for treatment measures (4).

-Treatment

Although its complex biological nature makes the management of oral mucositis a challenge, many strategies are used by oncologists to minimize the adverse effects of anticancer treatment, including dose reduction and other both therapeutic and preventive measures. The present study was designed to examine the main treatment options for oral mucositis due to chemotherapy found in the scientific literature.

## Material and Methods

An exhaustive search was made of the PubMed-Medline, Cochrane Library and Scopus databases, crossing the key words “oral mucositis”, “prevention” and “treatment” with the terms “chemotherapy” and “radiotherapy” by means of the boolean operators “AND” and “NOT”. We included human studies and review articles published in Spanish or English over the last 10 years. Opinion articles, series involving fewer than 5 cases, and studies using radiotherapy as sole or concomitant treatment were excluded. A total of 268 articles were obtained, of which 96 met the inclusion criteria (51 clinical trials, 3 cohort studies, 5 cross-sectional studies, 4 case series and 33 reviews).

## Results

Many strategies are used by oncologists to minimize the adverse effects of cancer therapy, including dose reduction and the prescription of other therapeutic and preventive options (2,7). An account is provided below of the main strategies used for the management of oral mucositis due to chemotherapy, described in the literature over the last 10 years.

-Oral hygiene protocols

Most of the published articles report some benefit from the use of oral hygiene protocols for the prevention of oral mucositis, since the resulting decrease in microbial presence reduces the risk of secondary infections (1,3,5,6). The study published by Hickey *et al.* (3) in patients with testicle cancer compared a group of individuals who received dental treatment before chemotherapy, along with instructions on oral and dental hygiene, versus a group in which no oral hygiene protocol was used. A 29% decrease in the prevalence of moderate oral mucositis was observed in the former group. Other studies have obtained similar results (5-7). Although the effects of such measures in preventing mucositis are questionable (5-7), most authors suggest that oral hygiene protocols (careful brushing and the use of dental floss and rinses) can reduce the duration and severity of mucositis, as well as contribute to prevent bacterial colonization in the context of mucositis (3,5,6).

-Antimicrobial agents

Regarding the use of chlorhexidine, the results found in the literature are contradictory. Nashwan (8) conducted a review of clinical trials using chlorhexidine in pediatric patients scheduled to receive chemotherapy. Of the 5 studies that met the inclusion criteria, four reported an important preventive effect in relation to the development and severity of oral mucositis. However, other studies indicate that chlorhexidine is not effective in reducing the severity of mucositis (2,6), and it has even been described that rinses with saline solution or bicarbonate may be equally effective as well as less costly (2,6-8). The systematic review conducted by Potting *et al.* (9) found no beneficial effects of chlorhexidine in comparison with rinses in the form of sterile water or physiological saline solution. Similar results were obtained in a systematic review published by Worthington *et al.* (10), who disadvised the use of chlorhexidine for the prevention of mucositis, since it was not found to be more effective than placebo. However, rinses with povidone iodine reduced the severity of oral mucositis by 30% compared with sterile water rinses (9). In contrast to other antiseptics, povidone iodine does not damage the oral mucosa. Studies have also been made of iseganan hydrochloride, though no significant effects in terms of the prevention of mucositis have been recorded (6,7,10). The review published by Rubenstein *et al.* (7) concluded that the use of antimicrobial agents for the prevention of oral mucositis is not justified, since a degree of benefit could only be expected in patients with late stage ulcerative mucositis, when the risk of bacterial overinfection is greater.

-Antiinflammatory agents

Benzidamine possesses antiinflammatory, analgesic, anesthetic and antimicrobial properties, and has been used for both the prevention and treatment of oral mucositis, with contradictory results (2,10,11). Other antiinflammatory drugs used for the prevention of oral mucositis due to chemotherapy are misoprostol rinses, histamine in gel format, and the intravenous / intramuscular administration of immunoglobulins (11,12). However, the study published by Dueñas-Gonzalez *et al.* (12) recorded an increased incidence and severity of mucositis in the group treated with misoprostol in tablets versus the placebo group. Another more recent study by Lalla *et al.* (11) likewise recorded no beneficial effect with the use of misoprostol in rinses (200 µg in 15 ml of water), in a series of 22 patients. Diphenhydramine rinses and mesalazine in gel format have also been studied, and although the results of the different publications suggest that such products may be effective, further research is needed to determine their true efficacy (12).

-Cytoprotective agents

Amifostine is believed to act by suppressing reactive oxygen species (ROS), which play a key role in the etiopathogenesis of oral mucositis. However, as a result of either methodological deficiencies or the use of a small sample size, the different studies have not found amifostine to reduce the duration or severity of mucositis induced by chemotherapy (2,6). As described by the literature, another less widely used cytoprotective agent with little impact upon the management of oral mucositis due to chemotherapy is sucralfate – the side effects of which include nausea and other gastrointestinal disorders such as rectal bleeding (5,6). Another suggested treatment is the topical application of prostaglandins E1 (misoprostol) and E2 (used to protect the digestive mucosa), with contradictory results (2,10,11). Vitamin E (α-tocopherol) is an antioxidant that can limit ROS-mediated tissue damage, and thus lessen the severity of mucositis during cancer therapy (2,3,13). El-Housseiny *et al.* (13) evaluated the effect of topical versus systemic vitamin E in patients with oral mucositis due to chemotherapy, and concluded that the topical application of 100 mg of vitamin E twice a day results in disappearance of the mucositis lesions. However, Sung *et al.* (14) did not find the prophylactic use of vitamin E to lessen the appearance of oral mucositis in children treated with doxorubicin. Further studies are needed, since this substance has been shown to be effective in treating established lesions, but does not prevent the development of new lesions (3,13,14). Glutamine has been used for both the prevention and treatment of mucositis, administered via the oral route, as rinses, and via the enteral and intravenous routes. One of the studies included in the review published by Rubenstein *et al.* (7) examined the effect of glutamine administered via the parenteral route in 24 patients with metastatic colorectal cancer treated with 5-fluorouracil, and recorded a significant decrease in mucositis and gastric ulcerations in the group of patients administered glutamine versus placebo (*p*<0.01). However, other authors have obtained contradictory results, including Pytlik *et al.* (15), who not only found glutamine to be ineffective in preventing mucositis but also suggested that the drug could worsen mucositis and even increase the risk of tumor relapse. The randomized, double-blind, controlled multicenter phase III clinical trial carried out by Peterson *et al.* (16) in breast cancer patients subjected to chemotherapy examined the efficacy of Saforis® (oral glutamine) at a dose of 2.5 g/5 ml administered three times a day versus placebo, and recorded a significant decrease in the incidence and severity of oral mucositis in the patients treated with Saforis®. Another drug used for the prevention of mucositis is irsogladine maleate, which is not marketed in Spain. Only one study is found in the literature, involving the administration of 4 mg/day of irsogladine via the oral route during 14 days from the first day of the chemotherapy cycle in patients treated with 5-fluorouracil. The drug was seen to significantly reduce the incidence of oral mucositis versus the control group (17).

-Biological response modifiers

In the treatment of cancer, growth factors are indicated for reducing the duration of neutropenia in patients with non-myeloid malignancies subjected to chemotherapy, and for accelerating myeloid recovery in patients subjected to bone marrow transplantation. Many studies, mostly published before the year 2004 (thus causing us to conduct an independent search), have found that rinses containing granulocyte colony-stimulating factor (G-CSF) and granulocyte macrophage colony-stimulating factor (GM-CSF) may significantly reduce the duration and severity of mucositis (18-23). In this regard, in the study of Crawford *et al.* published in 1999 (18), involving patients diagnosed with lung cancer and treated with cyclophosphamide, etoposide and doxorubicin, the percentage of patients who developed oral mucositis was seen to be lower in the group treated with subcutaneous G-CSF than in the control group (53% versus 70%, respectively). The study published by Katano in 1995 (19), involving G-CSF via the subcutaneous route, and the article published by Karthaus in 1998 (20) with G-CSF rinses, have obtained similar results. However, the randomized, controlled clinical trial carried out by Patte *et al.* in 2002 (24) did not find the administration of G-CSF via the subcutaneous route to be effective in preventing oral mucositis. Regarding the use of GM-CSF, the study published by Chi *et al.* in 1995 (23) found the systemic administration of GM-CSF in patients with head and neck cancer subjected to chemotherapy (5-fluorouracil and cisplatin) to reduce the severity and duration of oral mucositis. Similar data have been obtained by other studies with the use of GM-CSF rinses, with reduction of the severity, morbidity and duration of oral mucositis induced by chemotherapy (Ibrahim in 1997 (21) and Hejna in 2001 (22)). However, Cartee *et al.* in 1995 (25) did not find rinses containing GM-CSF to reduce the appearance of oral mucositis in patients with breast cancer subjected to chemotherapy (5-fluorouracil, adriamycin and methotrexate). Palifermin is a human truncated recombinant form of keratinocyte growth factor (KGF) produced by recombinant DNA technology in Escherichia coli. It is indicated in patients with hematological malignancies subjected to myeloablative therapy, which is associated to a high incidence of severe mucositis, since the drug stimulates epithelial cell proliferation and increases the thickness of the non-keratinized layers of the oral and gastrointestinal mucosa – thereby reducing the incidence, duration and severity of mucositis. Palifermin is administered via the intravenous route at a dose of 60 µg/kg/day during three consecutive days before and after myelosuppressive therapy, for a total of 6 doses. The third dose is administered 24-48 hours before bone marrow suppression (2,10,26). According to the reviewed literature, the administration of palifermin at doses between 1-180 µg/kg/day reduces the incidence and severity of oral mucositis ( Table 1) (26-30). The most frequent adverse reactions affect particularly the skin and oral mucosa, with dysgeusia, paresthesia, hypertrophy of the oral mucosa and tongue papillae, color changes of the oral mucosa, rash, pruritus, erythema and hyperpigmentation of the skin, among other alterations (26,30). Other undesirable effects include cough, rhinitis and arthralgia. These problems are usually mild or moderate in intensity, appear in the last three days of treatment, and according to some studies do not require interruption of the drug (2,6,26,30).

**Table 1:**
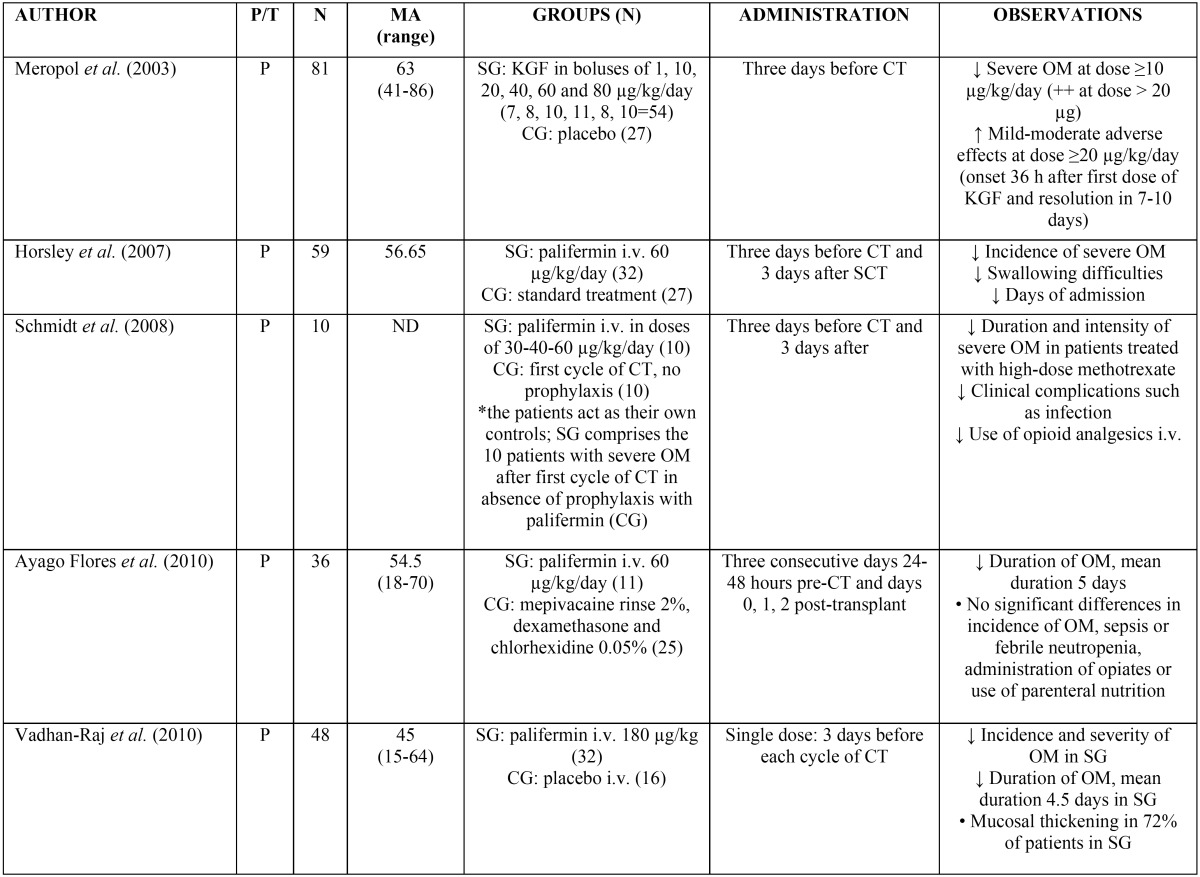
Use of palifermin in the management of oral mucositis (26-30). P/T: prevention / treatment of oral mucositis; N: number of patients; EM: mean age in years; ND: not declared; SG: study group; CG: control group; i.v.: intravenous; CT: chemotherapy; SCT: stem cell transplantation; OM: oral mucositis; KGF: keratinocyte growth factor-palifermin.

-Physical therapies (cryotherapy and laser)

The topical application of ice (cryotherapy) on the oral mucosa has been shown to offer benefit in the prevention of oral mucositis in some patients receiving chemotherapy. The precise underlying mechanism is not clear, though as mentioned by Mahood *et al.*, cryotherapy is believed to induce local vasoconstriction – thereby reducing oral mucosal blood flow and exposure of the mucosa to the cytostatic agent, with a consequent decrease in direct toxicity (2). Since the half-life of 5-fluorouracil is short (5-20 minutes), different studies have found that the application of cryotherapy during 5-10 minutes before administration of the drug, 15-35 minutes during administration, and up to 30 minutes after administration, significantly reduces mucositis (31-39). Studies have also been made in patients administered conditioning treatment with high-dose melphalan, with good results (35,36). However, the results obtained in patients administered other cytostatics such as methotrexate, etoposide, cisplatin, mitomycin, edatrexate and vinblastine are inconclusive (6,36,37,39) ( Table 2). Furthermore, cryotherapy is not indicated in patients treated with certain chemotherapeutic agents such as oxaliplatin, since acute neurological manifestations may develop in the form of mandibular stiffness and laryngopharyngeal dysesthesia (6). Phototherapy with low power laser has also been used for both the prevention (10) and treatment of oral mucositis due to chemotherapy (38,40-43). Different studies have described a decrease in the incidence and severity of mucositis, apparently due to acceleration of affected tissue regeneration and healing, thereby reducing the inflammation and pain (10,38). A number of authors support the use of low power laser for preventing oral mucositis in patients subjected to hematopoietic stem cell transplantation and scheduled for high-dose chemotherapy (with or without total body irradiation)(10,38,40-44). However, the clinical trial carried out by Cruz *et al.* (44) in pediatric patients yielded no evidence that the use of low power laser affords increased benefits ( Table 3).

**Table 2:**
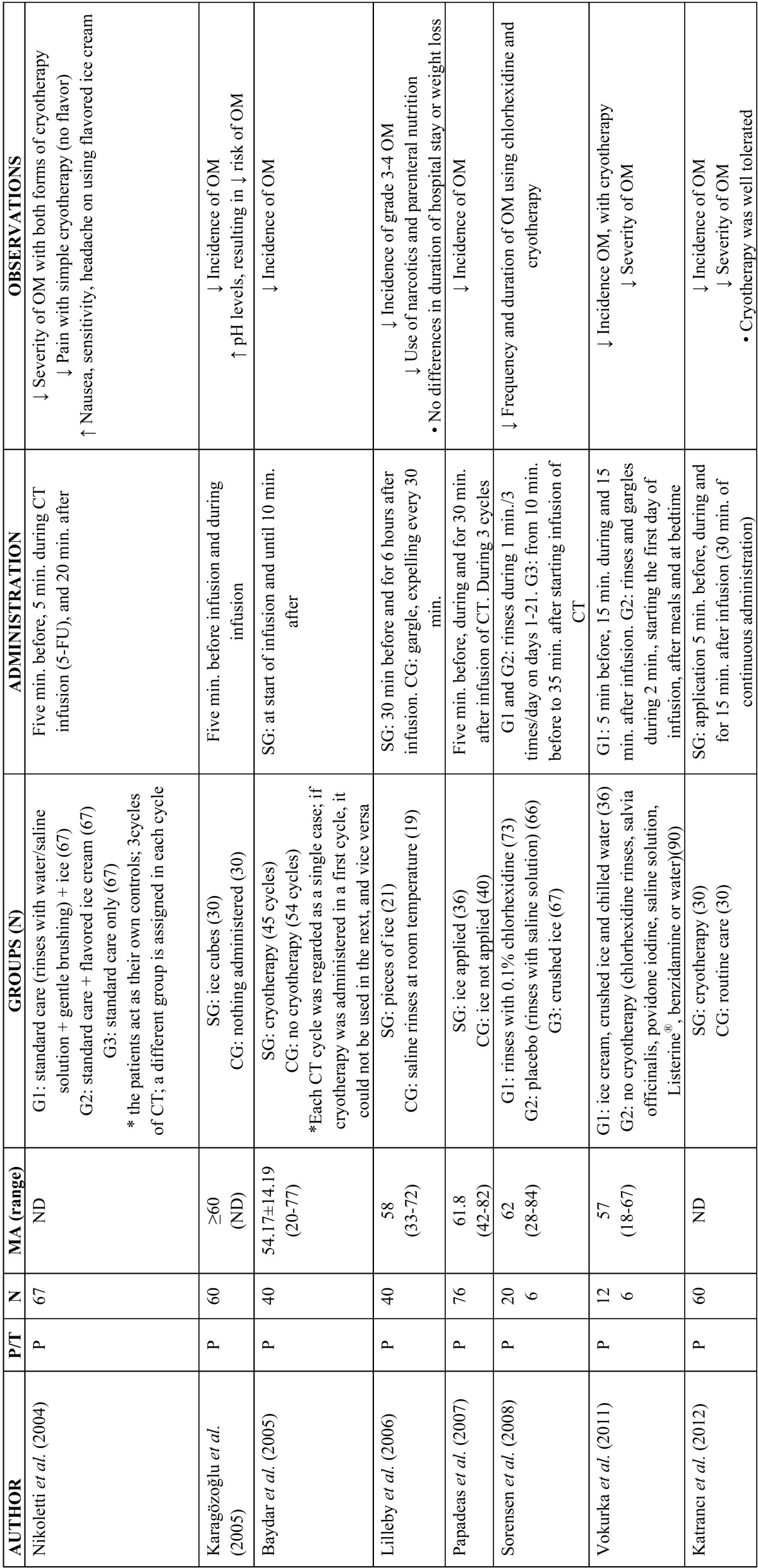
Use of cryotherapy in the management of oral mucositis (31-37,39). P/T: prevention / treatment of oral mucositis; N: number of patients; EM: mean age in years; SG: study group; CG: control group; CT: chemotherapy; OM: oral mucositis; min.: minutes; 5-FU: 5-fluorouracil.

**Table 3:**
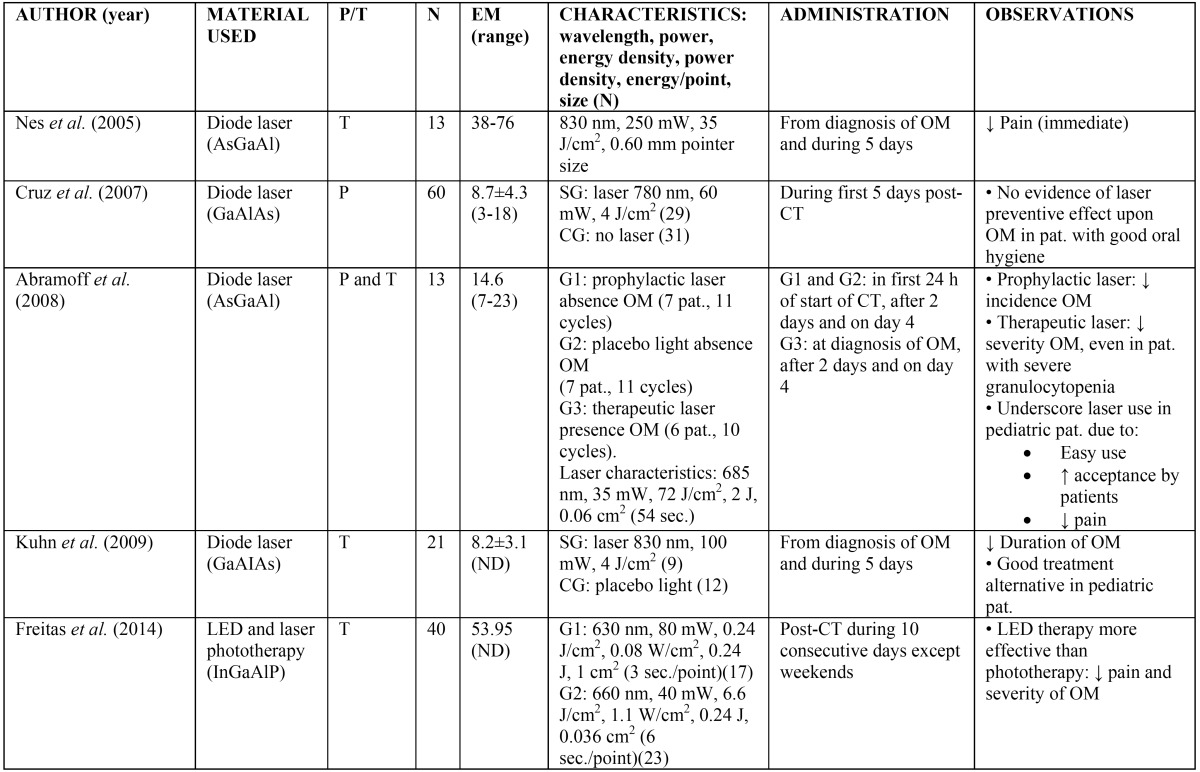
Use of laser therapy in the management of oral mucositis (40-44). P/T: prevention / treatment of oral mucositis; N: number of patients; EM: mean age in years; ND: not declared; pat.: patient; G: group; SG; study group; CG: control group; CT: chemotherapy; OM: oral mucositis. *This study is divided into two clinical trials; reference here is to the first trial, since the second included patients that received radiotherapy.

-Anesthetics and analgesics (management of pain)

Although no drug has been shown to successfully eliminate mucositis, the management of the pain symptoms with anesthetic solutions (diphenhydramine, viscous xylocaine and lidocaine) and potent analgesics such as morphine rinses, the application of sublingual methadone or fentanyl patches, could afford relief from the oral discomfort and improve patient quality of life (6,7,10,38). A so-called “magic mouthwash” has been described, containing variable amounts of diphenhydramine, viscous lidocaine, bismuth subsalicylate and corticosteroids, with the purpose of affording pain relief and lessening the inflammation. However, some studies have not recorded significant improvement of the pain (6,7,10,38). The application of capsaicin and the use of colchicine rinses have also been described as treatments for pain associated to mucositis (6,38). According to the review published by Worthington *et al.* (10), there is no evidence that patient controlled analgesia is better than continuous infusion, although less opioid is administered per hour and the duration of pain is shorter. Nevertheless, different studies recommend patient controlled analgesia in place of continuous infusion or administration supervised by the nursing personnel (7,10,38).

-Other agents

Allopurinol administered in rinses, pieces of ice or via the systemic route has been studied for the prevention of mucositis in patients receiving chemotherapy with 5-fluorouracil or methotrexate, though the results obtained are inconclusive (6,10,12,38). On the other hand, propantheline reduces salivation and may thus lessen oral mucosal exposure to chemotherapeutic agents that are excreted in saliva (2). According to some publications involving small sample sizes and with shortcomings in terms of design, propantheline can reduce mucositis associated to the administration of etoposide or the combination of different chemotherapeutic agents (ifosfamide, carboplatin and etoposide) in patients subjected to autologous hematopoietic stem cell transplantation (2). Some studies advocate the use of Caphosol® rinses for the prevention and treatment of mucositis (10,38,45). These rinses are composed of two aqueous electrolytic solutions in separate containers - a phosphate solution (Caphosol A) and a calcium solution (Caphosol B) - which when combined in equal volumes form an oversaturated solution of calcium and phosphate ions that humidify and lubricate the oral mucosa. Waśko-Grabowska *et al.* (45) found the administration of Caphosol® rinses to reduce the incidence, severity and duration of mucositis in patients treated with BEAM regimens (carmustine, cytarabine, etoposide and melphalan), in contrast to the group treated with melphalan 200. Arbabi-kalati *et al.* (46) administered 220 mg of zinc sulfate daily in capsule form to patients receiving chemotherapy, and observed a decrease in the intensity of mucositis. However, the incidence in the control group was similar. Some studies have examined the usefulness of honey and propolis in the management of mucositis, in view of their antibacterial and regenerative properties. Although further research is needed, involving a larger number of patients (6,7,10,38), honey may be a valid alternative for improving the symptoms and shortening the duration of mucositis.

## Discussion

The management of oral mucositis is a challenge, due to its complex biological nature (2,5,6,10,38). Although the scientific literature describes a number of management strategies, the data available to date are heterogeneous and inconclusive (5,10,38).

The Cochrane Library has published a series of reviews on the prevention of oral mucositis produced by radiotherapy and/or chemotherapy (2000, 2003, 2006, 2007, 2010 and 2011). The latest update conducted by Worthington *et al.* (10) concluded that only 10 interventions (aloe vera, amifostine, cryotherapy, granulocyte colony-stimulating factor (G-CSF), intravenous glutamine, honey, keratinocyte growth factor, laser irradiation, polymyxin / tobramycin / amphotericin (PTA) antibiotic tablet / paste, and sucralfate) offer some benefit in terms of the prevention or reduction of mucositis associated to cancer therapy. Of these 10 interventions, cryotherapy, palifermin and sucralfate showed statistically significant benefit in preventing or reducing the severity of mucositis. It should be noted that the use of cryotherapy was exclusively investigated in patients with hematological malignancies subjected to chemotherapy or stem cell transplantation; palifermin in patients subjected to radiotherapy, stem cell transplantation, chemotherapy or a combination of these treatments; and sucralfate in patients subjected to radiotherapy.

Since it is safe, inexpensive and generally well tolerated, cryotherapy is one of the most commonly used interventions for the prevention of oral mucositis, particularly in patients receiving treatment with short half-life chemotherapeutic agents such as 5-fluorouracil, edatrexate and melphalan (3-5,10,31-38). Different studies in patients treated with 5-fluorouracil have shown that the administration of cryotherapy 5-30 minutes before and for 20-30 minutes (even up to 6 hours, according to some studies) after the 5-fluorouracil bolus dose significantly reduces oral mucositis (31-38). The latest update on the guides developed by the mucositis study group of the Multinational Association of Supportive Care in Cancer and the International Society of Oral Oncology (MASCC/ISOO), published in 2014, recommend the use of cryotherapy in patients administered 5-fluorouracil in bolus form, and suggest its use in patients treated with high-dose melphalan as conditioning therapy for hematopoietic stem cell transplantation - independently of whether total body irradiation is used or not (5). However, due to the lack of evidence in the reviewed studies, it has not been possible to establish guidelines for patients receiving other chemotherapeutic agents (5).

At present, the recommendations of different clinical guides include prophylaxis with palifermin, in order to reduce the incidence and duration of mucositis in patients with hematological malignancies subjected to hematopoietic stem cell transplantation and prior myeloablative therapy, only when the latter includes high doses of chemotherapy and total body irradiation (5-7,38). Howe-ver, there are also standard chemotherapy regimens that do not require stem cell support, which can be associated to severe mucositis. Nevertheless, the high cost of palifermin and the efficacy data published to date advise the conduction of studies involving larger patient samples, in order to establish the impact of palifermin upon other variables such as the incidence of mucositis, sepsis or febrile neutropenia. Likewise, pharmacoeconomic studies are needed to facilitate decision making in selecting efficient preventive treatment for mucositis (28).

The literature contains a number of studies that have evaluated the effects of laser therapy in patients receiving chemotherapy, with encouraging results (39-43), in contrast to the study published by Cruz *et al.* (44). The MASCC/ISOO clinical guide published in 2014 recommends treatment with laser involving specific characteristics (wavelength about 650 nm, power setting 40 mW, and treatment of each square centimeter during the required period of time at a tissue energy dose of 2 J/cm2) for the prevention of oral mucositis in patients receiving high-dose chemotherapy for hematopoietic stem cell transplantation with or without total body irradiation (5). In addition, research is needed to clarify the biological mechanism whereby laser irradiation is able to improve healing and lessen pain (38).

The literature describes a number of interventions for the prevention and treatment of oral mucositis. However, although there is evidence recommending the use of certain treatments, none of them have been clinically validated, and there is no gold standard for managing mucositis (6). Additional methodologically sound clinical trials with a sufficient number of patients are therefore needed in order to allow adequate analyses of subgroups according to the type of disease and the chemotherapeutic agent involved (5,38). Such trials in turn should be reported following the recommendations of the Consolidated Standard of Reporting Trials (CONSORT). Furthermore, as indicated by Worthington *et al.* (10), it would be useful for investigators to use a simple mucositis scale (scored from 0-4), such as those developed by the World Health Organization (WHO), the Radiation Therapy Oncology Group (RTOG), or the National Cancer Institute-Common Toxicity Criteria (INC-CTC), in order to facilitate comparison among the different interventions used in application to mucositis.
